# Revising Protein Corona Characterization and Combining ITC and Nano-DSC to Understand the Interaction of Proteins With Porous Nanoparticles

**DOI:** 10.3389/fbioe.2021.650281

**Published:** 2021-10-11

**Authors:** Alba Balmori, Romica Sandu, Daniela Gheorghe, Alina Botea-Petcu, Aurica Precupas, Speranta Tanasescu, David Sánchez-García, Salvador Borrós

**Affiliations:** ^1^ Grup D’Enginyeria de Materials (GEMAT), Institut Químic de Sarrià, Universitat Ramon Llull, Barcelona, Spain; ^2^ “Ilie Murgulescu” Institute of Physical Chemistry, Bucharest, Romania

**Keywords:** protein corona, nano-DSC, ITC, characterization, mesoporous silica nanoparticles (MSNs)

## Abstract

The exposure of nanoparticles (NPs) to biological fluids leads to the formation of a protein coating that is known as protein corona (PC). Since PC formation is influenced by the physicochemical properties of the nanoparticles, the understanding of the interplay of the factors that participate in this process is crucial for the development of nanomaterials as cell-targeted delivery vehicles. In general, it is accepted that the PC formation is a complex and dynamic process, which depends on the composition of the medium and the properties of the NP mainly size, shape, and superficial charge. Interestingly, although the interaction between the protein and the NP is essentially a superficial phenomenon, the influence of the roughness of the nanoparticle surface has been scarcely studied. In this work, the influence of superficial roughness and porosity has been studied with the aid of nanodifferential scanning calorimetry (nano-DSC) and isothermal titration calorimetry (ITC) using mesoporous silica nanoparticles (MSNs) as an NP model. The interaction process of the proteins with the NP surface was analyzed by ITC measurements, while the stability and denaturation of the proteins was monitored by nano-DSC. Thanks to the complementarity of these two techniques, a more complete insight into the PC formation on the pores has been accomplished.

## Introduction

In recent years, significant efforts have been drawn focusing on designing nanoenabled drug delivery and biocompatible therapeutically active agents. These systems offer many advantages in drug delivery, such as improved efficacy, biosafety, and stability of the drugs. (Hamidi et al., 2008; Mudshinge et al., 2011; Singh and Lillard, 2012; Balcells et al., 2019). However, all these advances are still far to be translated into clinic. Many factors account for this drawback. However, one of the main issues is the interaction of the nanoparticles (NPs) with the biological medium. When NPs enter the blood stream, they immediately adsorb proteins on their surface. (Lynch and Dawson, 2008; Gao and He, 2014). This coating on the NP’s surface is known as the protein corona (PC). Depending on the affinity of the protein absorbed, this coating is known as the “hard” corona when the affinity is high and the proteins are tightly bound or “soft” corona consisting of loosely bound proteins, probably interacting with hard corona proteins. The nature of the PC determines the biological fate of the nanoparticle, such as cellular uptake, circulation time, bioavailability, and even toxicity, and it is crucial to achieve the goal as a drug delivery system. (Ferrari, 2005; Castillo et al., 2013). In this sense, recently, the importance of the PC in the biodistribution (Sarparanta et al., 2012) or targetability of the mesoporous silica nanoparticles (MSN) (Rosenholm et al., 2010) as well as the biocompatibility (Tang et al., 2012), among other effects has been shown. Hence, a precise understanding of the parameters that govern the PC formation is of critical importance for the design of efficient nanovehicles for drug delivery. (Hadjidemetriou et al., 2015; An and Zhang, 2017).

The structure of PC is determined by the kinetic and equilibrium binding of protein with a nanoparticle, which depends on the medium composition and on the nanoparticles’ properties, such as size, surface charge, surface composition, and functionality. (Lundqvist et al., 2011; Mahmoudi et al., 2013; Ritz et al., 2015; Lee, 2017). However, despite the extensive studies and the known influence of the NP physicochemical properties in the formation of the PC, only a few reports pay attention and focus on the influence of the NP roughness and pores in the formation of the PC, which is one of the keys in the final physiological response of the nanoparticle. (Ma et al., 2014; Gao et al., 2016).

Herein, we have developed a new method to study on how the porosity of the nanoparticle affects the formation of the PC. For this purpose, nano-DSC and ITC techniques will be used. As model NP’s, mesoporous silica nanoparticles (MSNs) have been chosen due to their wide application on drug delivery and their synthetic versatility (Ibrahim et al., 2010; Tu et al., 2016). Human serum albumin (HSA) Peng et al. (2013) and lysozymes have been used as protein models because of their presence in the human plasma and their different sizes (69 and 34 Å, respectively). The methodology proposed in this article could be used in general to a more complete characterization of the protein corona formation process with a great variety of nanoparticles. (Vidaurre-Agut et al., 2019).

## Results and Discussion

First, to gain insight into the influence of the MSNs’ pore size on the protein corona formation, the ITC method has been used to analyze the formation process of the protein corona. ITC is a powerful technique, which can measure interaction heats with a great accuracy, and allows a full description of thermodynamic parameters arising from the binding interaction (such as enthalpy change, affinity, and the number of protein molecules bound per particle) (Nasir et al., 2015).

**FIGURE 1:**
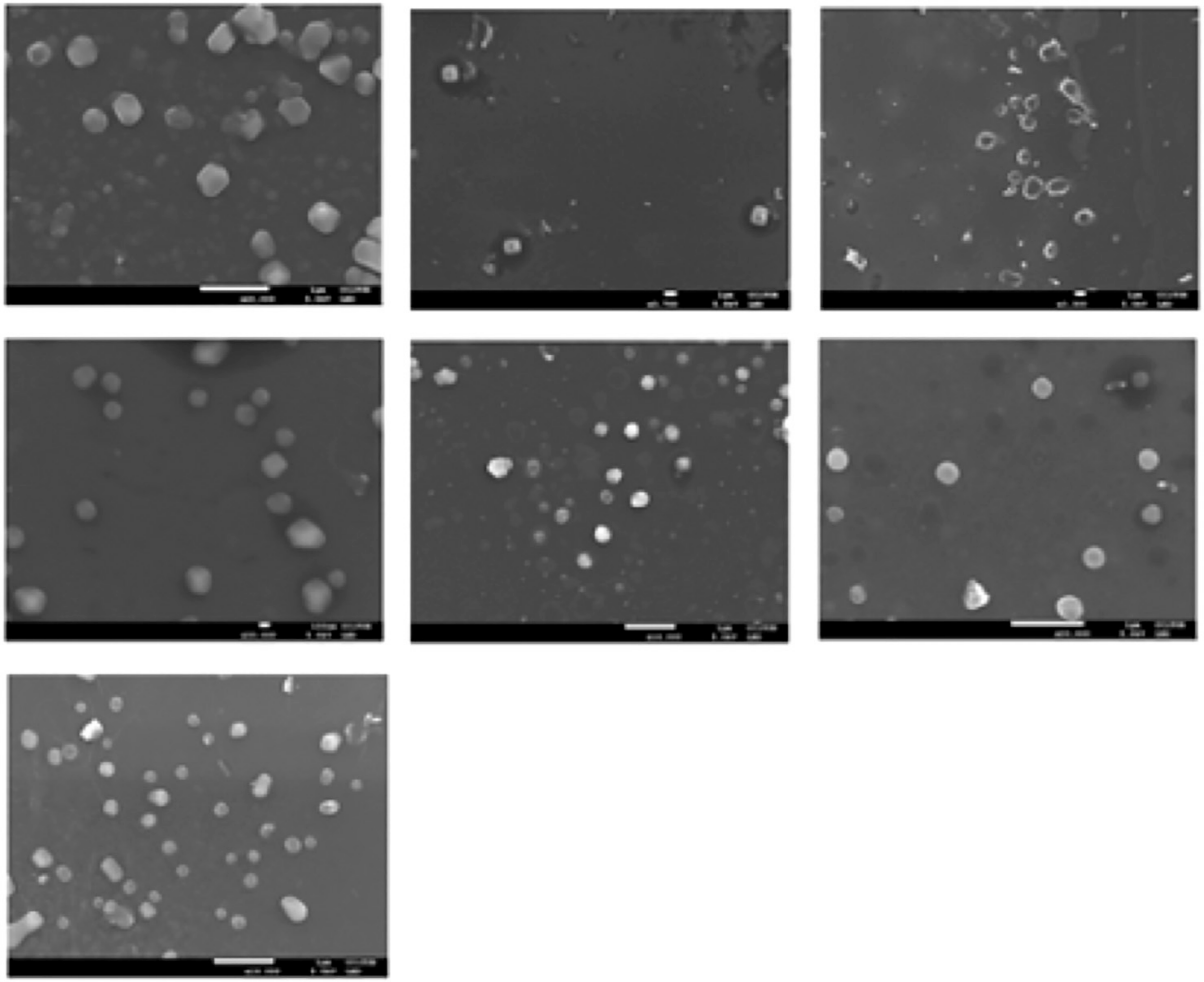
SEM images. From the left to the right and from the top to the bottom: S1, S2, S3, S4, S5, S6, S7, and S112

The nanoparticles chosen to be studied present enough variety in terms of size, pore size, and aspect ratio to demonstrate the generality of the method (Table 1). Thus, suspensions of four types of nanoparticles with different pore sizes S1, S3, S6, S7, and nanoparticles without pore (S112) used as a control were titrated with a solution of HSA in water (see Table 1 for the description of the analyzed MSNs). The experiments were performed at 37°C to simulate the physiological medium. In this article, we propose to use this technique for extracting information about the dynamics involved in the interaction between the porous surface and the protein (Figure 1).

**TABLE 1:** Nanoparticles tested and their main structural features.

References	Size (Å)	Porous size (Å)	Aspect ratio	Zeta potential (mV)
S1	180	38.8	1.16	28.8
S3	360	38.3	1.08	26.9
S4	217	34.2	1.2	27.7
S5	430	28.8	1.19	31.7
S6	180	36.0	1.04	28.3
S7	260	30.8	1.44	26.2
S112	320	No pores	1.00	−3

The size and aspect ratio have been determined from SEM micrographs. The aspect ratio is calculated from the average dimensions of the particles. Regarding the relationship between aspect ratio, size, and curvature, in general, when the aspect ratio is close to 1 (spheres), the smaller the nanoparticle is, the higher the curvature is. In Figure 1, the SEM images are shown.

At the beginning of the titration, an endothermic signal emerges with positive values of heat. This signal is ascribed to the formation of the complex HSA-NP, which indicates this interaction is an enthalpically unfavorable, entropically driven process. In this process, the disruption of the water molecules bound to the NP surface is energy dependent. The increase in the entropy of the system, due to the release of the highly ordered solvent molecules from the interface to the medium, would compensate for the unfavorable enthalpy contribution.

**FIGURE 2:**
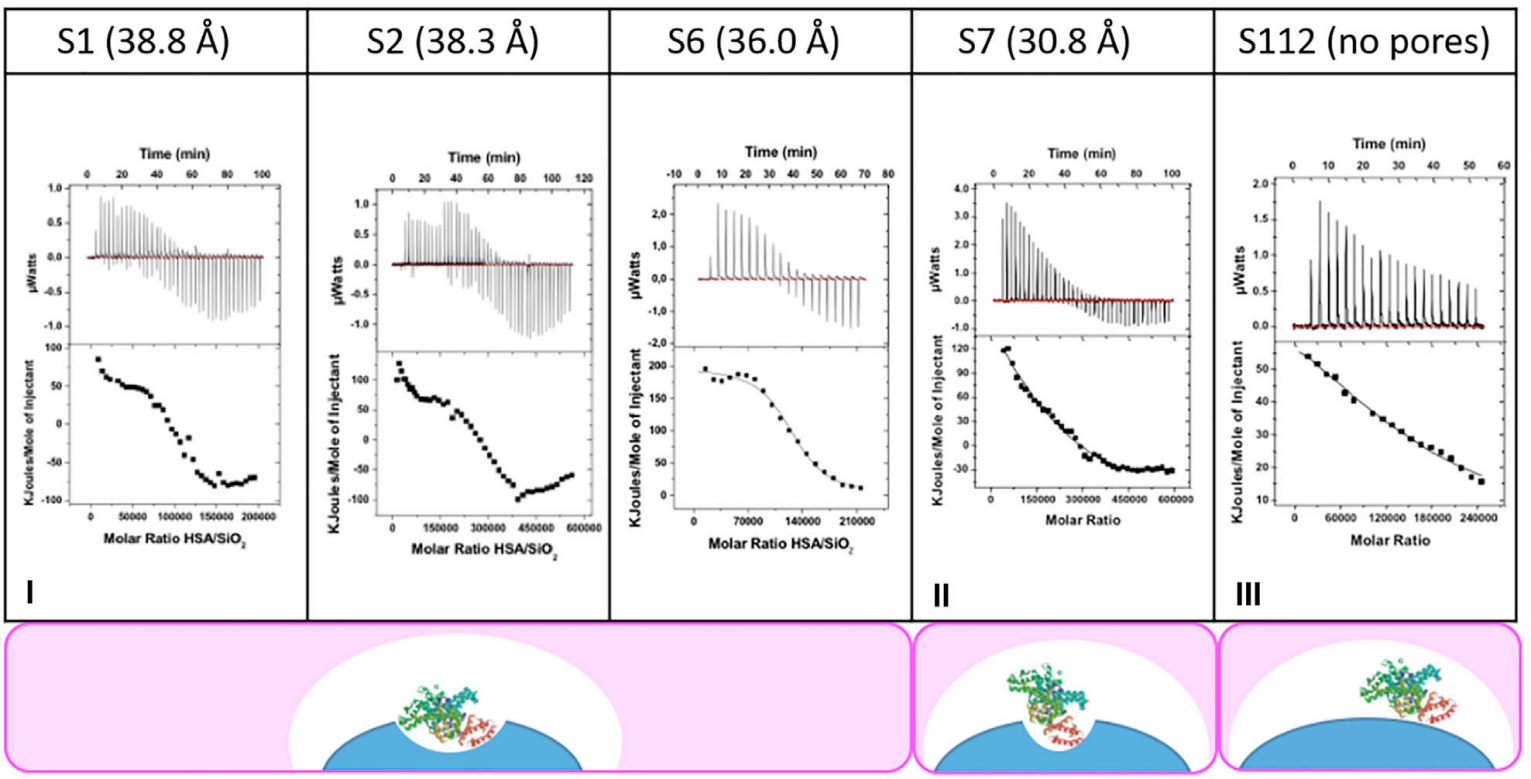
ITC’s thermograms of the different nanoparticles studied (S1, S3, S6, S7, and S112) with different pore sizes (38.8, 38.3, 36.0, 30.8 Å, and no pore, respectively) titrated with HSA solution.

The gradual addition of HSA renders the appearance of exothermic peaks. It is reported in the literature that the curvature of the nanoparticle can account for such exothermic response in the presence of proteins. However, there is no correlation between the aspect ratio of the nanoparticles studied (S1, S3, S6, S7, and S112) (see Table 1) and the area increase of the exothermic process at higher concentrations. Thus, these results cannot be explained only by the curvature effect. (Zhang et al., 2017; Geras, 2008). Hence, we hypothesize that the exothermic peaks vary with the pore size. The larger the pore size is, the higher the exothermic peak is (see Figure 2). Pores’ width enough to fit and stabilize the protein would play a more important role in the formation and the structure of the protein corona on nanoparticles (Loregian et al., 2007; Winzen et al., 2015). In fact, the sample of the compact nanoparticle, with no pore, confirms the abovementioned behavior since there is only the surface effect and no exothermic peaks are observed (Figure 2E).

Collectively, thermograms, as shown in Figure 2, demonstrate that the adsorption of the protein is a two-step process. First, the protein approaches the surface of the MSNs and binds loosely in its native state at random positions on the surface of the nanoparticle. Then, gradually, the protein undergoes conformational changes to form noncovalent protein–nanoparticle bounds that help to stabilize the protein into the pore. These interactions lead to stronger adsorption, which would be responsible for the observed exothermic transitions. (Wang et al., 2008).

To give insight into the exothermic contribution registered by ITC and to study how the NP–protein interaction affects the stability of the protein, nano-DSC measurements were carried out. In this article, we suggest the use of this analytical technique to obtain information about protein denaturation. In brief, during protein–NP incubation, proteins from the medium are adsorbed onto the NP surface forming the protein corona. As stated before, once the proteins interact with the surface of the nanoparticles, they may undergo conformational changes. In this sense, it can be said that the structural nature of a protein is different when the protein is forming a part of the protein corona or if it is unbound. Thus, it is possible to differentiate the binding state of the protein related to the proteins’ stability. It is described (Nierenberg et al. (2018), Shah and Singh (2019)) that usually proteins forming the protein corona are less stable than the free proteins. Moreover, it has been reported that the binding of proteins to surfaces often induces significant changes in the secondary structure. (Saptarshi. et al., 2013). For instance, serum albumin adsorbed on MSNs surfaces showed a rapid conformation change at both secondary and tertiary structure levels. (Walkey and Chan, 2012). We propose to monitorize the overall protein stability (thermal denaturation) by nano-DSC to give insight into the protein–nanoparticle interaction in the formation of protein corona on MSNs.

**FIGURE 3:**
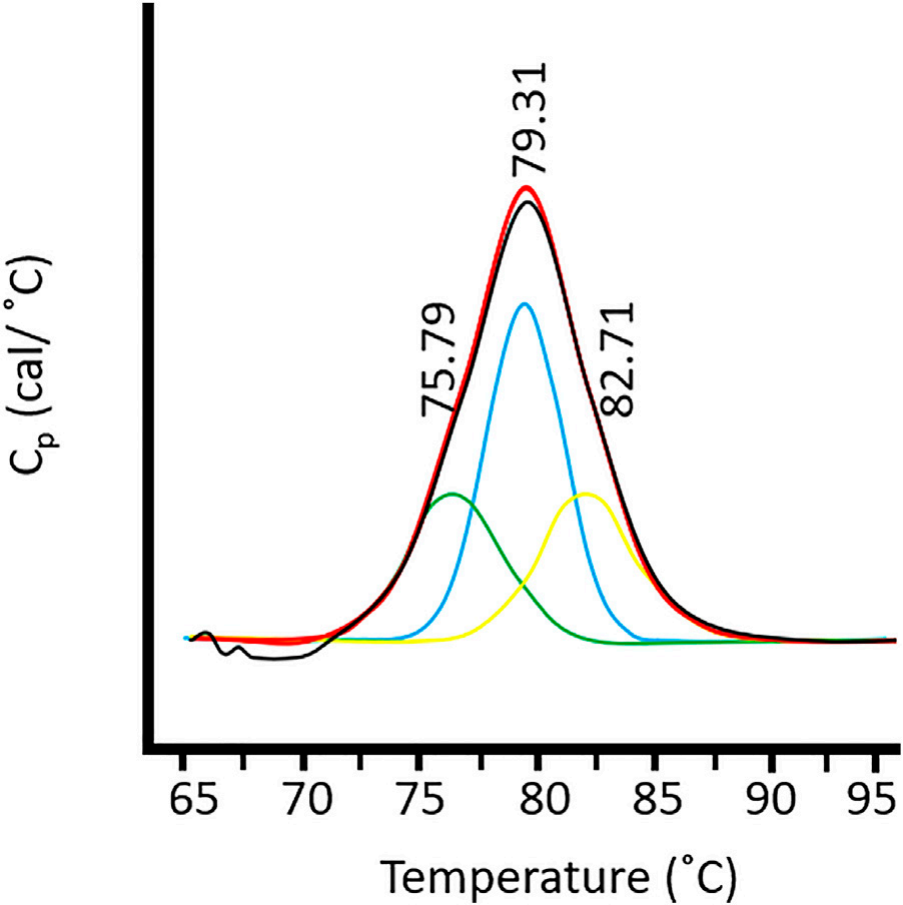
Deconvolution of the thermal unfolding signal of HSA in the presence of S1 MSN (*T*_*m*1_ = 75.79°C, *T*_*m2*_ = 79.31°C, and *T*_*m3*_ = 82.71°C).

The aim is to determine the transition midpoint, *T*_m_, of the free protein and compare it with the value obtained in the presence of porous nanoparticles (Lang and Cole, 2014). This transition midpoint, *T*_m_, is considered the temperature in which 50% of the protein remains in its native conformation, and the rest is denatured. This temperature can be assigned to the peak of the nano-DSC transition observed (see Figure 3). Higher *T*_m_ values would be representative of more stable proteins (Bai et al., (2011)).

We hypothesized that the signal obtained from the nano-DSC measurements can be deconvoluted into three Gaussian curves, which represent three different protein states (Setyawati et al., 2015): protein far from NP, proteins coating the surface of the NP, and proteins interacting with the pores (Chiu and Prenner, 2011; Bampi et al., 2016). First, there is a population of proteins that are only weakly ligated to the NP generating a corona of almost unaffected proteins and thus almost no change occurs in their *T*_m2_. Second, there is also a population of proteins interacting with the NP surface, thus more exposed to the disruption of their native form, resulting in less stable proteins (lower *T*_m1_). The third population can be ascribed to the process of occlusion of the pores by the proteins. This allows the formation of a highly stable protein corona composed of the proteins internalized in the pores (*T*_*m3*_). Thus, a high temperature curve corresponding to the denaturation of these proteins could be assigned in the deconvolution of the nano-DSC signal.

Figure 3 presents the thermogram of HSA denaturation in the presence of S1 MSN. Three different transitions can be observed through the deconvolution of the DSC thermogram. As explained before, the first deconvolution peak, which appears at a lower temperature, corresponds to the albumin destabilized by its interaction with the S1 surface. The second and third peaks described above, namely, albumin weakly ligated to the nanoparticle (central deconvolution peak) and that corresponding to the albumin inside the pores) can be clearly identified. It can be concluded that, through the deconvolution of nano-DSC plots, the influence of the two effects and the surface and pores on the protein corona formation on MSNs nanoparticles could be analyzed and quantified.

**FIGURE 4:**
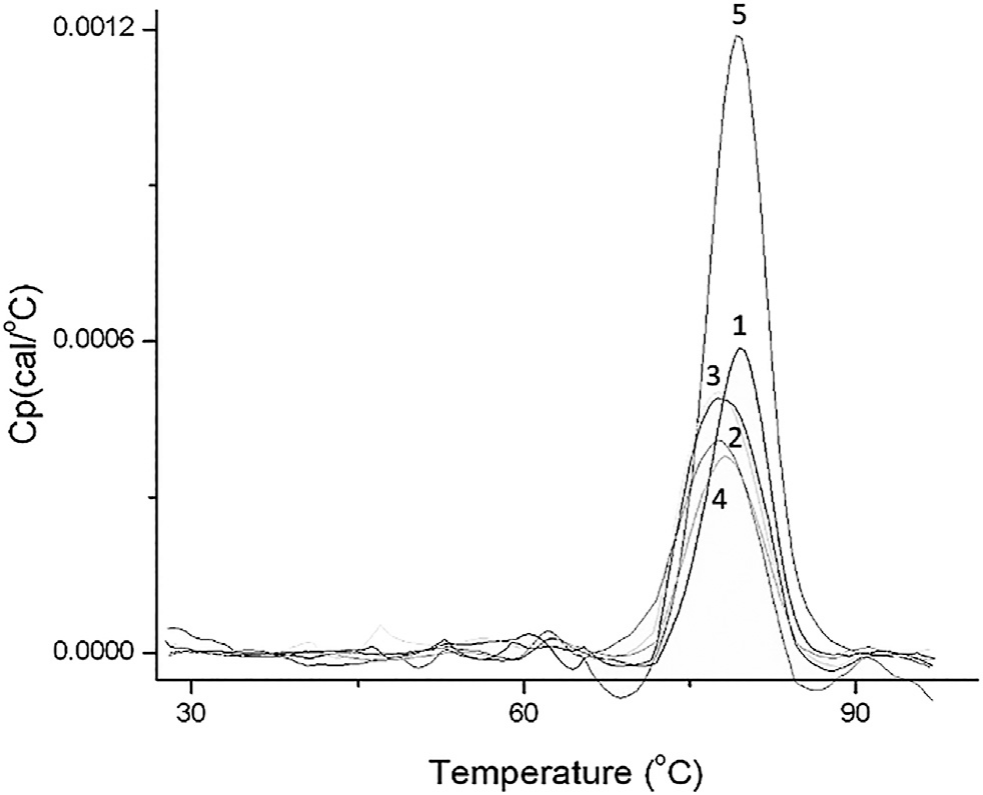
Nano-DSC plot of HSA incubated with nanoparticle S1 for different incubation times: (1) HSA; (2) 5 min of incubation; (3) 30 min, (4) 10 h; and (5) 24 h.

In order to prove this hypothesis, the signal for HSA thermal denaturation after different incubation times—5 minutes, 30 minutes, 10 hours, and 24 hours with S1—was registered (Figure 4). It can be observed that, after longer times of incubation, the DSC signal maximum shifts to a higher value and presents a higher amplitude. We see this as a confirmation that HSA (because their size) fits inside the NP pore. It means that, over time, the protein molecules adopt conformations which allow fitting in the pore. It has been corroborated that, in the first stage, the protein forms a layer with a random conformation on the surface of the nanoparticle, and in the second stage, it acquires different conformations that cause its entrance to the pore. Finally, after longer incubation times (24 h), an external layer of weakly bounded HSA is also formed, increasing only the second peak of the deconvoluted signal of the DSC plot (protein cloud formation) (Nierenberg et al., 2018).

**FIGURE 5:**
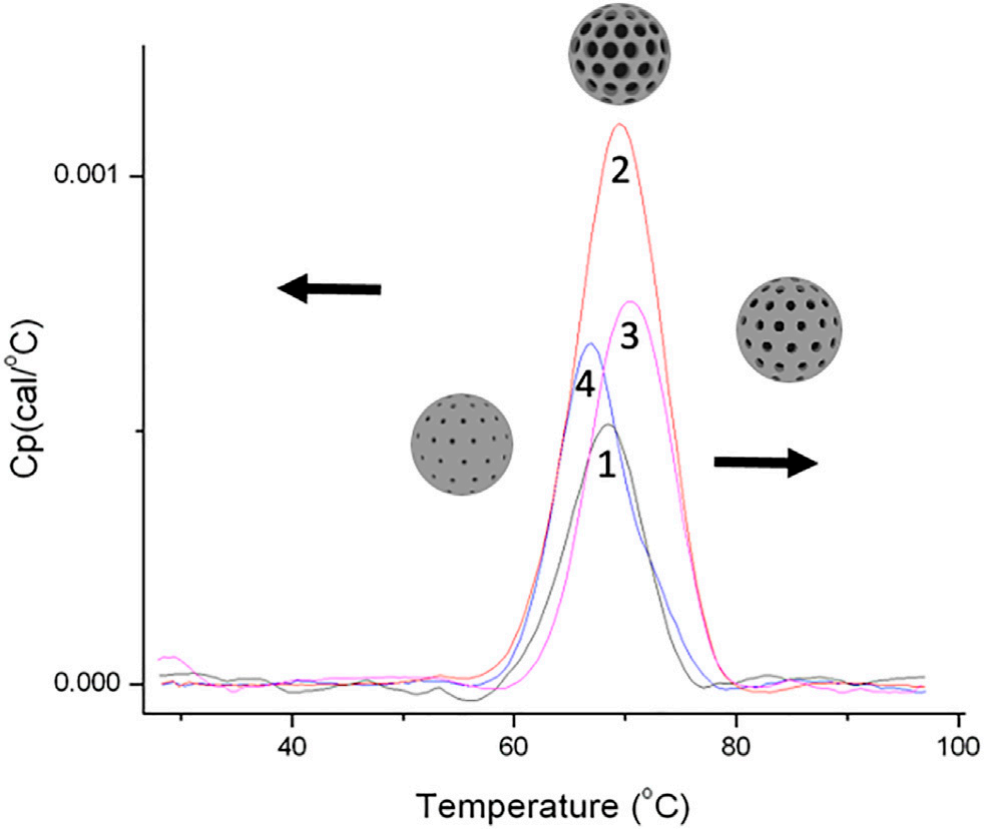
Nano-DSC plot of the interaction between lysozyme and three nanoparticles with different porous sizes: (1) free lysozyme; (2) S1 38.8 Å; (3) S4 34.2 Å; and (4) S5 28.8Å.

To confirm the role of the pore size in the nano-DSC results, lysozyme (34 Å, and 14.3 kDa) was exposed to nanoparticles with different pore sizes (S1 with a pore size of 38.8 Å, S4 with 34.2 Å, and S5 with 28.8 Å). The results obtained are shown in Figure 5. It can be seen how in S5 MSN the lysozyme is not able to enter the pore, and practically only on the surface it had an effect in the PC formation (the peak shifts to the left as we expected). S4 presents a pore size where the lysozyme fits smoothly. This fitting confers stability to the protein and the peak shifts to the right. Finally, in S1 MSN, the pore is clearly wider than the protein and it can easily enter into the channels. In this case, the PC formation is influenced by the surface and the pore at the same time (the base of the peak widens).

**FIGURE 6:**
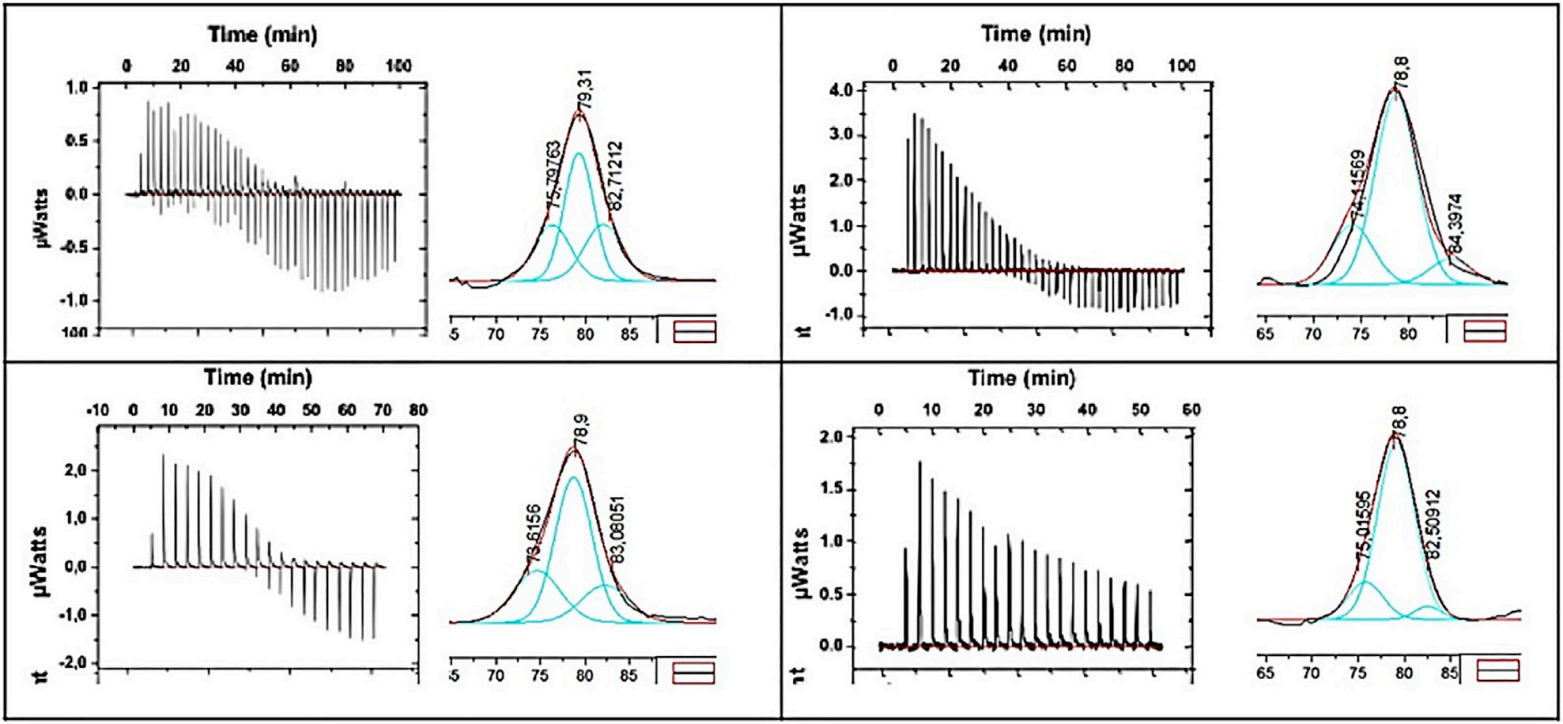
Comparison between nano-DSC and ITC for S1, S6, S7, and S112 MSNs.

The joint analysis of the two techniques allows the understanding of the building process of the PC. However, ITC provides information on the dynamics of the hard protein corona formation inside the pores, and DSC gives insight into the nanoparticle effect on the protein structure stability. The ratio between the exothermic contribution (corresponding to the negative heat values obtained by integrating the individual peaks in the exothermic part) and the total heat in absolute values (obtained by integrating all the individual peaks by the instrument software) suggests how favorable the interaction of the protein with the pores of the nanoparticle is. The DSC studies indicate that this ratio correlates with the integral of the Gaussian curve at higher temperature, which is ascribed to the heat change due to the structural changes underwent by the protein to fit into the pores. The results of the comparison are presented in Figure 6. As can be seen, the results are very similar and, in our opinion, confirm that both techniques are complementary and allow to study the interaction between proteins and pores in MSNs.

As demonstrated, the combination of ITC and nano-DSC provides a quick methodology to study the PC formation process with porous MSN. From a practical point of view, it would be possible to foresee and tailor the formation of coronas by adjusting the dimensions of the pores.

To test this hypothesis in a practical example, a proteomic assay was made. The composition and concentration of the PC were identified in a detailed proteomic analysis by LC-MS. Nanoparticles were incubated in FBS for 24 h by following the usual protocol, and the formed PC were analyzed. On the other hand, it was also analyzed that the PC formed on MSN incubated with HSA during 24 h followed by the incubation with FBS during another 24 h. The samples selected were S1 and S5, whose pores size is 38.8 Å and 28.8 Å, respectively. The results obtained are summarized in Table 2.

**TABLE 2:** The three most abundant proteins in PC: BSA (bovine serum albumin), TRFE (serum iron transport protein transferrin), TRFE2 (telomeric repeat-binding factor 2), HSA (human serum albumin), ApoAI (apolipoprotein A1), and Glyco (glycoprotein).

*S1*				S5			
FBS		HSA + FBS		FBS		HSA + FBS	
BSA	31.4%	HSA	32.2%	ApoAI	34%	ApoAI	23.9%
TRFE	24.5%	BSA	13.6%	BSA	14.5%	HSA	18.7%
TRFE2	23.8%	ApoAI	12.2%	Glyco	10.9%	BSA	13.9%

The three most abundant proteins in each PC were compared. The molecular weights of these proteins are as follows: BSA 69.2 kDa, TRFE 77.6 kDa, TRFE2 77.7 kDa, HSA 69.3 kDa, ApoAI 30.3 kDa, and Glyco 38.4 kDa (all of them are abundant in plasma). From these data, it can be observed that the PC formed in S1 incubated with FBS, the most abundant protein was albumin, while in S5 was ApoA1. As anticipated, the natural formation of a PC in FBS yields a PC composed by proteins which can be fitted into the pore. In the same way, the proteins that form the PC in S1 had an MW of 69.2, 77.6, and 77.7 kDa, as opposed in S5 30.3, 69.2, and 38.4 kDa. On the other hand, nanoparticles incubated 24 h previously with HSA were allowed a PC formed entirely for HSA. During the following 24 h in FBS, the PC undergoes a replacement of the HSA for other proteins. S1 maintains 32.2% of HSA, being the majority, while in S5 was only 18.7%. In the case of S5, the majority of the proteins were the new ApoAI which fits in the porous nanoparticle due to its size.

## Conclusions

The interaction of a model protein with the porous surface of MSN has been studied using ITC and nano-DSC. We propose to use the combination of the two techniques to obtain valuable information about the protein corona formation on MSNs. In our approach, ITC provides information on the dynamics of the hard protein corona formation inside the pores. On the other hand, DSC gives insight into nanoparticle effect on the protein stability.

This assessment on the PC is a powerful tool for developing artificial protein coronas. It is envisioned that artificial coronas can be tailored to endow porous carriers with selectivity and longer circulation times by coating the nanoparticles with the proper proteins. Furthermore, with the aid of ITC and nano-DSC, proper coatings can be engineered using proteins and even smaller nanoparticles to prepare efficient delivery systems, thereby avoiding premature drug release. From an analytical point of view, it has been demonstrated that porous nanoparticles can play a role in the diagnosis of diseases by the proteomic analysis. The presented methodology would help to develop and optimize such methodologies.

## Materials and Methods

### Mesoporous Silica Nanoparticles Synthesis

To a solution of 0.2 g of CTAB (cetyl trimethyl ammonium bromide) in 48 ml of H_2_O was added a solution of ammonia AM and was allowed to rise to B°C during 30 min at C rpm. Then, to this solution 1 ml of TEOS (Tetraethyl orthosilicate) at D ml/h was added dropwise using an automatic injector, followed by the addition of E ml of APTES ((3-aminopropyl)triethoxysilane). The resulting solution was stirred at the same temperature for additional 3 h. Solid samples were collected via centrifuging at 13,000 rpm for 13 min, washing and dispersing with deionized H_2_O and EtOH, twice. Surfactant was removed by extraction in acidic ethanol, 0.5 ml of HCl in 20 ml of EtOH at 65°C for 24 h. Again, samples were collected via centrifuging at 13,000 rpm for 13 min, washing and dispersing with deionized H_2_O and EtOH, twice. Finally, the NPs were lyophilized (See Table 3 for A; B; C; D; and E values).

**TABLE 3:** Synthesis conditions for the Silica nanoparticles used in the study.

Sample	A (M)	B (°C)	C (rpm)	D (ml/h)	E (ml)
1	0.2	60	1,100	2	3.85
2	0.2	80	1,100	4	20
3	0.2	80	1,100	2	12.8
4	0.2	60	1,100	7	20
5	0.5	60	1,100	4	12.8
6	0.5	80	1,100	7	3.85
7	0.2	60	550	7	12.8
8	0.5	60	550	2	20
9	0.2	60	550	4	3.85
101	0.5	80	550	7	12.8
102	0.5	80	550	7	3.85

### Synthesis of Silica Nanoparticles (S112)

A solution of 3 g of PVP and 30 ml of 1-pentanol was ultrasonicated for 2 h at room temperature. Then, 3 ml of absolute ethanol, 0.84 ml of ultrapure water, and 0.2 ml of 0.18 M sodium citrate dihydrate (0.5294g/10 ml). The solution was shaken by hand, and 0.675 ml of ammonia (30% p/p) was added into the solution. The solution is shaken again, and finally, 0.3 ml of TEOS was added. Then, the solution was aged, without stirring for 24 h. Solid samples were collected via centrifuging at 201 g (1,500 rpm) for 15 min; subsequently, the as-synthetized samples were washed and dispersed with deionized H_2_O and EtOH, twice. Finally, the nanoparticles were lyophilized.

### SEM Microscopy

Samples were observed using an FE SEM JEOL JSM-7001. Prior to that, they were ultrasonically dispersed in H_2_O at a concentration of 1mg/10 ml and then 1/20. The samples were deposited on an amorphous, porous carbon grid. In order to make the samples conductive, a layer of graphite was deposited on the MSN surface.

### Pore Size Quantification

The pore size of the nanoparticles was determined by the adsorption/desorption of nitrogen using a Micromeritics Gemini V Series Surface Area Analyzer. In brief, 17 mg of each sample was lyophilized at 0.05 mBar, −0.8°C, during 24 h inside BET tubs to remove the solvent at low temperature. Pore size distribution curves were obtained from the analysis of the adsorption portion of the isotherms using the BJH (Barrett–Joyner–Halenda) method.

### Differential Scanning Calorimetry (DSC)

The protein thermal stability and the thermodynamic parameters of HSA denaturation (both free in solution and in the presence of MSNs) were estimated by using a nano-DSC (TA Instruments) equipment. Before DSC measurements, the HSA-MSN solutions (concentrations 4 mg ml^−1^ HSA and 0.2 mg ml^−1^ MSNs) were prepared by incubating MSNs in aqueous protein solution for 24 h at 37°C. The measurements were done in the temperature range of 25–98°C, with a scanning rate of 1°C min^−1^, and at the pressure of 2 atm. The partial heat capacity contribution from water and MSNs in water was measured independently and subtracted from those of the individual protein and protein adsorbed onto MSNs, respectively. The calorimetric data were corrected for the calorimetric baseline between the initial and the final state by using a sigmoidal baseline from NanoAnalyze software. The transition temperature (*T*_m_) and thermodynamic parameters represented by the heat capacity change (∆*C*_p_) and enthalpy change (∆*H*) of HSA in the presence of MSNs were evaluated. DSC traces were deconvolved using OriginPro 9.0.0.0 (64-bit) SR2 software. The deconvolution was made fitting here using Gaussian functions.

## Isothermal Titration Calorimetry (ITC)

The calorimetric measurements were performed using an ITC200 Microcalorimeter (MicroCal). The MSN dispersions and HSA solution were prepared using MilliQ water (Millipore).The MSN dispersions were sonicated using a probe sonicator (SONOPULS HD 3100, Bandelin, Germany) for 10 min with 10% amplitude and 7.192 kJ energy. All the probes were degassed for 10 min under vacuum with a MicroCal ThermoVac degasser, prior to their use in ITC experiments. Injection parameters were specific for each sample: The concentration of the nanoparticles varied between 2 mg ml^−1^–3 mg ml^−1^, depending on the surface area and pore size of MSN, in the sample cell, and a solution of HSA (7 mg ml^−1^) was drawn into the syringe. The reaction started by a titration of 39 μL HSA solution in 240 μL MSNs suspension. The time between the titration steps was 150 s and was chosen to reach an equilibrium state. During all experiments, the temperature of the ITC device was kept constant at 37°C. The nanoparticles dispersions inside the sample cell were permanently stirred at 500 rpm. Sample data were analyzed by Origin® software.

### Protein Isolation for MS

Protein corona isolation was performed as described elsewhere with minor alterations. In brief, MSNs resuspended in DMEM were centrifuged at 16,000 g for 1 h at 4°C to separate the excess proteins from the cell media. The supernatant was discarded, and the pellets were resuspended in a PBS–Tween20 0.05% (w/v) solution. The pellets were washed twice with the PBS–Tween20 solution and a fourth time with PBS alone by centrifuging at 201 g (1,500 rpm) for 1 h at 4°C. A solution of 8 µL of NuPAGE 4X LDS and 4 µL of 500 mM dithiotheitol (DTT) is added to the pellets and mixed gently to resuspend each pellet. The solution is incubated at 70°C for 1 h and centrifuged for 15 min at 16,000 g and the 12 µL supernatant is used for the next step. 95 µL of an aquous solution of ammonium bicarbonate at 100 mM, 5 µL of acetonitrile, and 5 µL of DTT 100 mM were added to the protein pellets and left at 80°C for 45 min. The solution was left to cool down until it reached the room temperature, and 11.5 µL of 500 mM iodoacetamide in ammonium bicarbonate (pH 7–8) was added and incubated in dark conditions and room temperature for 1 h. 460 µL of pure acetone prechilled at −20C was added, and the solution was left at −20°C for protein precipitation. Proteins were centrifuged at 16,000 g for 10 min at 4°C, the supernatant was discarded, and pellets were air-dried before resuspending them in 100 µL of ammonium bicarbonate at 50 mM (pH 7-8). 4 µL of a protease mixture of trypsin and lysine was added at the concentration indicated by the supplier and incubated overnight at 37 °C. Finally, the protein digestion was stopped by 10 min incubation at −80°C, and samples were kept at −20°C until MS analysis.

### MS

All mass spectrometric experiments were performed using an Orbitrap Fusion Lumos mass spectrometer (Thermo Scientific). The MS was coupled to an EASY-nLC 1,200 system (Thermo Scientific). The HPLC mobile phases were 96.1:3.9 water/acetonitrile with 0.1% formic acid (A) and 20.0:80.0 water/acetonitrile with 0.1% formic acid (B). The flow rate was 300 nL/min, and the following gradient was used for each run; 0% B for 5 min, 0–5% B in 10 min, 5–30% B in 150 min, 30–90% B in 30 min, and 90% B for 10 min. The tryptic peptide digests were diluted by a factor of ten with 0.1% formic acid, and aliquots of 1 µL of the diluted digests were injected onto and separated by a PepMap RSLC, C18, 3 μm, 100 Å, a 75 μm × 150 mm EASY-Spray column (Thermo Scientific). Electrospray ionization was performed at a voltage of 1.9 kV.

## Data Availability

The raw data supporting the conclusions of this article will be made available by the authors, without undue reservation.
