# Supplementary figures and images for: Revising Protein Corona Characterization and Combining ITC and Nano-DSC to Understand the Interaction of Proteins With Porous Nanoparticles
Source: Front Bioeng Biotechnol. 2021 Oct 11;9:650281. doi: 10.3389/fbioe.2021.650281 (PMC8542777; doi:10.3389/fbioe.2021.650281)

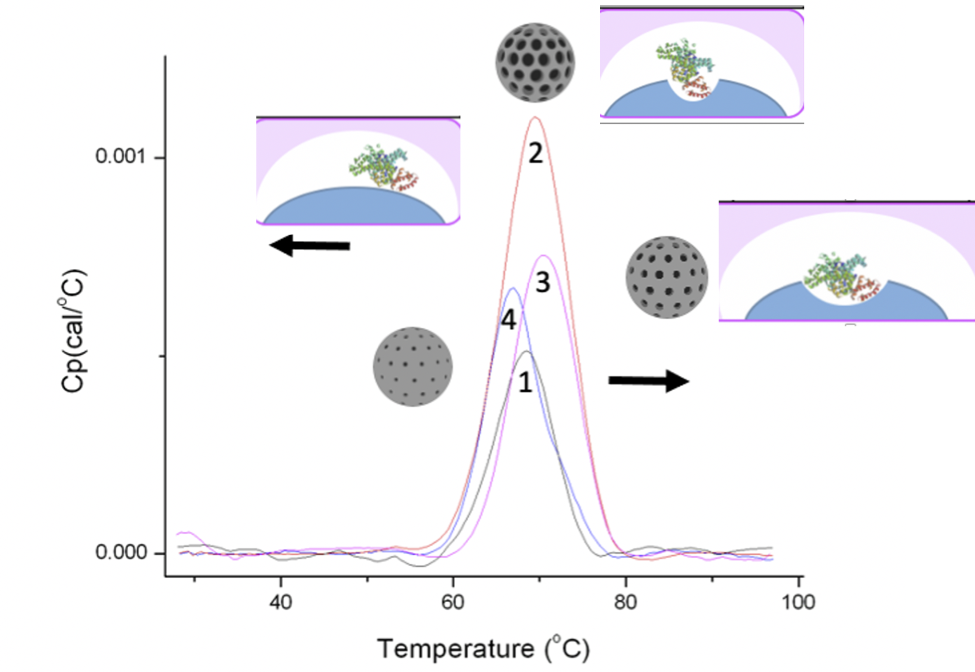

Supplement: Supplementary file 1 [file Image1.TIFF]
